# Towards a Practical Climate Ethics: Combining Two Approaches to Guide Ethical Decision-Making in Concrete Climate Governance Contexts

**DOI:** 10.1080/21550085.2023.2236504

**Published:** 2023-08-01

**Authors:** Anthony Voisard, Ivo Wallimann-Helmer

**Affiliations:** Department of Geosciences, University of Fribourg Environmental Sciences and Humanities Institute, Fribourg, Switzerland

**Keywords:** Climate ethics, climate justice, methodology of mid-level principles, principlism, environmental pragmatism

## Abstract

This paper discusses two approaches to climate ethics for practical reflection and decision-making in concrete local climate change governance. After a brief review of the main conceptual frameworks in climate ethics research, we show that none of these leading approaches is sufficiently context specific and pluralistic to provide guidance appropriate for concrete local climate governance. As alternatives, we present principlism as a methodology of mid-level principles and environmental pragmatism as an ethical approach. We argue that the two methodologies of principlism and pragmatism offer a new pluralistic framework that allows real-world conditions and contexts to be properly integrated into ethical analysis and decision-making in climate governance.

## 1. Introduction

Climate ethics is concerned with the just distribution of atmospheric resources, emission rights, and climate responsibilities in climate governance. Mainstream approaches to climate ethics emphasize the fundamental role of normative considerations in climate policy. In spite of a growing number of scholars criticizing the lack of a practical focus, frameworks and tools for guiding hard governance choices on the ground and supporting ethical decision-making by practitioners, administration, and governance actors remain underdeveloped to date (André & Bourban, 2016; Bourban, 2014; Gardiner & Weisbach, 2016; Green & Brandstedt, 2021; Jamieson, 2014; Light, 2011; Voisard, 2020). Moreover, the often highly academic tone of discussions on climate ethics fails to engage policymakers, practitioners, local stakeholders and, in some cases, researchers from disciplines other than environmental ethics, political theory, or other conceptual backgrounds.

As a result, climate ethics approaches are not yet integrated into practice and environmental governance as well as they might be. This is in stark contrast to how bioethics is integrated into the medical establishment, the development of engineering ethics in practice, and the awareness of research ethics in the scientific community. As a first step in spanning this gap, we discuss two approaches to climate ethics for practical reflection and decision-making in climate change governance and show how they can complement each other as methodologies for guiding practical ethical decision-making in climate governance in various local contexts. We claim that combining these two approaches enables a new pluralistic framework to analyze and decide ethical conflicts in concrete climate governance contexts.

The paper proceeds as follows. After a brief review of the most prominent approaches in climate ethics research, we argue that these approaches are insufficiently context specific and pluralistic to provide guidance appropriate for concrete local climate governance. We present two more context sensitive approaches: principlism as a methodology of mid-level principles and environmental pragmatism as an ethical approach. At the core of both methodologies lies the idea that normative considerations and their importance depend on the specific context to be analyzed. Principlism relies on a pre-defined set of principles that need to be specified and weighted depending on context. Environmental pragmatism is a philosophical approach that claims that collective inquiry is the key to understanding ethical conflicts. We discuss the intersections of principlism and pragmatism, their possible tensions, and their complementarity. We argue that both methodologies together offer a more contextualized and pluralistic ethical framework than conventional normative approaches in climate ethics. This allows them to properly integrate the real-world conditions and concrete challenges of local climate governance.

## 2. Three Typical Approaches to Climate Ethics

Several normative models have been used to inform ethical reflection on the implications of climate change governance. The most prominent theories in climate ethics research draw on well-established normative theories in moral philosophy: consequentialism, deontology (e.g. Rawls-inspired approaches), and virtue ethics (Bernstein, 2015; Broome, 2012; Jamieson, 2014; Lamp, 2017; Lamp & Lane, 2016; Nussbaum, 2013; Singer, 2010). Consequentialist approaches to climate change focus on the maximization of well-being and the best avoidance of harm. Deontological theories of climate ethics most often foster the norms and rules necessary to proper differentiation of responsibilities within the international climate governance regime. Virtue ethics theories concentrate on the character traits to be developed by citizens, consumers, and producers in the face of the climate crisis.

In this section, we explain why the first two approaches are typical top-down approaches that try to deduce prescriptive conclusions for policy recommendations without sufficient sensitivity to context. For virtue ethics, we argue that despite its promising interest in concrete contexts, it lacks both normative guidance and resources for collective ethical deliberation. This is because it aims to operate without ethical principles or rules and places strong emphasis on individual action without considering collective inquiry.

### 2.1. Consequentialism and Deontology Models in Climate Ethics

Consequentialist reasoning on climate change typically focuses on the rational calculation of the net welfare of individuals with respect to the harmful effects of climate change and their reduction (Singer, 2010). This ethical approach can be broken down into several distinct theories. For example, John Broome, a leading figure of climate consequentialism, makes utilitarianism his ‘default’ ethical stance unless particular questions arise and challenge it (Broome, 2012, p. 114). Consequentialist reasoning typically holds that climate action is morally appropriate if it produces at least as much well-being as alternative actions and can avoid the most harm.

In a deontological approach, the violation of a right cannot be justified by any maximization of well-being: principles of justice take precedence over the calculation of the total welfare of a society (Rawls, 1999). From this point of view, the preservation of fundamental rights threatened by climate change, such as the right to life, the right to health, and the right to subsistence, has to be prioritized over any utility calculations (Caney, 2010). From a deontological perspective, climate change is typically approached through theories of distributive or corrective justice that aim to identify and justify a series of principles for guiding global climate governance^1^ (Baatz, 2013; Caney, 2005; Gardiner, 2004; Page, 2008, 2012; Shue, 1999). Usually, such climate ethics theories aim to specify how to understand the principle of common but differentiated responsibilities as a principle underlying global climate governance (Wallimann-Helmer, 2019b).

So far, the most prominent climate ethics frameworks have focused mainly on global considerations, such as climate change mitigation governance (Baer et al., 2010; Heyward & Roser, 2016; Maltais & McKinnon, 2015), although local, regional, and situated dimensions of climate ethics governance have attracted some recent research (Dietzel, 2022; Létourneau, 2021; McKendry, 2016; Voisard et al., 2020). Yet, the methodology typically employed by proponents of consequentialism and deontological approaches to climate ethics is a top-down methodology of applied ethics (Bufachi, 2004). Top-down approaches depend on deductive reasoning, which functions essentially through abstraction and thought experiments. This methodology usually starts from one or a set of abstract moral principles that are justified a priori. From these principles, the methodology then deduces the morally right kind of action by claiming that the decisions suggested are universally justified.

The top-down methodology to climate change governance can be criticized for at least three reasons. First, this sort of mechanical and unidirectional reasoning may fail to engage local stakeholders and political decision-makers due to its inability to suitably integrate information about the specific local governance contexts to which it is to be applied. The somewhat rigid methodological model of top-down approaches fails to resonate with the concrete particularities and needs of practitioners and their stakeholders and thus fails to support environmental decision-making adequately. A climate ethics framework that is more flexible and more in tune with real-world conditions is required to guide ethical decision-making.

Second, although consequentialism and deontology may be claimed to attach some importance to the specificities of climate action, these philosophical approaches are certainly not sufficiently specific to place and context. The criterion of universality is strongly attached to these ethical approaches, often without considering the full complexity of given contexts. In addition, this universalistic moral perspective clearly differs from the recommendations on climate change adaptation formulated in the IPCC’s fifth assessment report, which are more in line with a particularistic ethical framing (Voisard, 2021). Climate action and the diverse areas of climate policy require not a universalistic but a context-specific point of view if their ethical implications and challenges are to be properly addressed (Wallimann-Helmer, 2019b, 2021).

Third, the set and ordering of abstract moral principles offered by climate ethicists often do not grasp the fundamentally pluralistic nature of environmental practices and political decision-making. Climate ethics approaches are not yet integrated into practice and local environmental governance circles as well as they might be. The ethics approaches need to be more inclusive of norms and values that are not necessarily embedded in their ethical systems. This becomes particularly salient when top-down approaches and grand theories of the ‘invoke and apply’ type (Gardiner, 2021, p. 7) propose fixed frameworks for analyzing the three main pillars of global climate governance. This is claimed even though these pillars’ objects of responsibility vary greatly: in the case of mitigation governance, reducing emissions is key; in the case of adaptation and geoengineering strategies, the governance of risk is most important; and many believe that climate loss and damage action primarily demands compensation. These diverse objects of responsibility each imply different moral considerations, identifying not only various hierarchies and sets of principles but also various agents to act at various governance levels, whether collective, institutional, or individual (Wallimann-Helmer, 2016, 2019a).

It is this malaise of top-down approaches that Dale Jamieson discusses in one of the first articles on climate ethics: ‘if applied philosophy is to be worth doing, it must take real-world issues on their own terms rather than use them as props for philosophical discussion’ (Jamieson, 1990, p. 86). Over the past thirty years, very few climate ethics researchers have heeded this early recommendation. However, an alternative to the top-down model of applied ethics may emerge through a contextualist perspective in which sensitivity to place and context acquires new salience. A typical normative approach of this sort would be virtue ethics.

### 2.2. Virtue Ethics in a Dark Time: A First Alternative to Top-Down Approaches

Unlike consequentialists and deontologists, virtue ethicists suggest considering the character traits that should be developed in a role when determining the right actions to take. Proponents of virtue ethics tend to criticize how consequentialists and deontologists neglect the contexts, motivations, and emotions related to the reasons driving action. Rather than focusing on the consequences of an action or the ethical principles or rules to be followed, more attention should be given to the factors that may influence our intentions to perform specific actions. Virtue ethics calls for all individuals to engage in virtuous behavior (Jamieson, 2007). According to Jamieson, climate ethics is best conceptualized by emphasizing the need for a paradigmatic transformation of values or virtues, such as respect, responsibility, humility, and love (Jamieson, 2014). This implies that where the combination of seemingly insignificant actions can have a disastrous impact at large spatial and temporal scales, consumers and global citizens should be expected to nurture character traits favorable to climate action in the various contexts of climate governance.

Consequentialists and deontologists may criticize virtue ethicists for failing to provide general principles that help determine the right action to take in a given situation. Ethical theories often hold that the main purpose of ethics should be the formulation and justification of such principles, whereas virtue ethics focuses on context, and perhaps more importantly on the character traits and resources needed by actors in a given situation. In consequence, whereas top-down approaches can be criticized for being insufficiently sensitive to the various contexts of climate action and governance, virtue-ethical approaches seem to lack principles that guide decisions on what exactly to do in a specific conflicted context. Furthermore, virtue ethics can be criticized for its model of ethical reflection, which lacks resources to deal with collective ethical deliberation. Virtue ethics relies on the capacities of single agents and their ethical reflection in specific situations but typically does not provide resources for including various stakeholders in such processes.

By contrast, despite some situationist similarities with virtue ethics, philosophical pragmatism suggests articulating a broader analytical framework capable of envisioning democratic transformation to address social problems by engaging stakeholders in inclusive and practical deliberative processes. From a participatory governance perspective, this may mean providing recommendations and decision-making tools to help construct climate change adaptation strategies at the regional scales (Létourneau, 2019, 2021; Muccione et al., 2019). The tools arising from this type of pluralistic endeavor are not only designed to produce academic articles and scientific reports but also to transfer knowledge that provides the various local stakeholders with concrete and user-friendly instruments that support their collective planning of climate change adaptation.^2^

In the next section, we argue that a pragmatist approach in combination with a theory of mid-level principles to environmental ethics provides a better methodology for dealing with the challenges conventional climate ethics theories face. We argue that principlism provides contextualized principle-oriented guidance for environmental practitioners and environmental pragmatism provides a pluralist deliberative framework faithful to the richness and complexity of collective moral experiences. In our view, comprehensive and practice-relevant ethics can be obtained by combining both ethical perspectives.

## 3. Towards a Practical Framework for Climate Ethics

Climate ethics is arguably a prolific field of research that has made several conceptual advances in recent years. The challenge of reconciling efficiency with the demands of justice has led some scholars to suggest that ethical theorizing has no place in climate policy (Posner & Weisbach, 2010). However, the outright rejection of these theories risks responses to climate change that do not consider any moral reasoning at all (Gardiner & Weisbach, 2016). We agree with Gardiner that ethical concepts remain fundamental to guiding climate action and moral choices in our pluralistic and non-ideal world, where both democratic institutions and individuals are morally fallible in many ways. In the following sections, we argue for the relevance of two more context-sensitive and pluralist methodologies to mainstream climate ethics: principlism as the methodology of mid-level principles and environmental pragmatism as an ethical approach.

### 3.1. Principlism as the Methodology of Mid-Level Principles

As explained above, the application of principles proposed in the climate ethics literature can be criticized for ignoring the contextual scope of issues associated with the three pillars of global climate governance. If this is the case at the global level, such approaches face even more serious troubles with the messy nitty-gritty governance of concrete contexts, each of which involving its own ethical and political challenges. Arguably, this criticism would not necessarily mean developing new principles of justice for each context but instead specifying and weighting climate justice principles according to the particular areas of climate governance (Wallimann-Helmer, 2015, 2019a, 2021). As we show in this section, this is exactly what a principlist methodology suggests.

A decontextualized application of mainstream climate ethics principles is particularly inadequate for situated areas of climate action, such as adaptation to climate change (Wallimann-Helmer, 2016). Adaptation measures usually have to be implemented at local or regional levels, and ethical conflicts depend not only on the specificities of the contexts but also on the various values, moral rules, principles, goals, and interests that emerge in the ethical deliberations of the stakeholders engaged in climate adaptation. In principlism, balancing risks of flooding by building dams or keeping recreation areas open to the sea cannot effectively be decided top-down by applying abstract principles but only by specifying what abstract moral principles mean in a context and by weighting their relative importance to its ethical challenges.

However, most research in climate ethics does exactly the opposite. It develops general articulations of the rights and duties of the parties to a global climate governance regime. Even though the main approaches in climate ethics may be suitable for addressing the global problem of greenhouse gas reductions and financing climate adaptation in a global climate governance regime, they typically do not detail the various objectives, specificities, and orientations at the different levels of climate policy and in the diverse areas of climate action. In the area of climate adaptation, this means that even though these theories can establish general rules for differentiating who must provide climate adaptation finance and according to what criteria these resources must be distributed, they are of little help when deciding which particular climate adaptation projects to prioritize or how to define which residual risks should remain (Wallimann-Helmer, 2015, 2016).

Principlism as a methodology of applied ethics is most prominent in medical ethics. It starts from the basis that it is possible to define a core set of ethical principles that form a common morality in a specific context of practice and governance (Ashcroft, 2007; Beauchamp & Childress, 2013). This core set of principles determines how to decide ethical conflicts. Principlism holds that the core set of principles must be specified and weighted differently according to the context, leading to differing moral evaluations (Beauchamp, 2007; Wallimann-Helmer & Keller, 2018). One principle might be decisive in some contexts but not in others, whereas another principle might be decisive in exactly these other contexts. Such a core set of principles in climate change governance could be formed by the principles of just burden sharing prominently discussed in climate ethics (Gardiner et al., 2010; Page, 2008): the polluter-pays principle, the beneficiary-pays principle, and the ability-to-pay principle. However, in contrast to most theories in climate ethics, principlism neither claims that the understanding of these principles can be fully defined independently of the context at issue nor argues for a specific relation or hierarchy between these principles independent of contexts. The following discussion of three classical principles of climate ethics illustrates what this might mean.

The polluter-pays principle is a principle of justice by which moral agents should bear the burdens of combating climate change in proportion to their contribution to causing the problem. Expressed more simply, and quite intuitively, this principle implies that if you contribute to climate harm, you are responsible for contributing to its mitigation in proportion to your causing climate change. According to the logic of this principle, the financial burden of combating climate change should be decided primarily on the overall historical accumulation of GHG emissions. As a result, it is argued that the largest proportion of climate burdens should be shouldered by the largest historical polluters of GHGs per capita, such as those living in the United States and in Europe. However, applying this principle at more local levels might be understood differently. In these contexts, it might not be all historical pollution that is relevant to assigning responsibilities but a specification of this principle that only considers emissions for consuming luxury goods. If pollution happened long in the past, the polluter-pays principle might not be the most important principle by which to solve the problem. Other principles might be evaluated as weighing more heavily.

In such situations, the beneficiary-pays principle could be such a principle. According to this second principle, the costs of dealing with climate change should be distributed in proportion to the benefits that have been derived from emissions. This principle implies that even if third parties are not directly responsible for contributions to climate change, they may nevertheless be required to compensate the victims of its negative impacts. Although industrialized countries have derived benefits from various economic, social, political, and scientific structures, it may appear unfair for some countries or some of their members to inherit the burden of responsibility from their ancestors, because these ancestors were often unaware of the future effects of their actions on the climate, and they did not have the opportunity to consult their descendants before taking the actions that are deemed harmful today. Even though this principle might be helpful in contexts where the beneficiaries of pollution are not the polluters, benefitting in the context of climate change might mean different things and hence demand different specifications of this principle: benefits can be derived from emitting, from changing climatic conditions, and from favorable climate policy (Lenzi et al., under review). Furthermore, in contexts such as climate adaptation, any link to emissions might be of secondary concern (Wallimann-Helmer, 2016).

The ability-to-pay principle suggests that the burdens of climate action ought to be shared according to the various abilities to contribute to climate measures. This principle of distributive justice does not necessarily imply the attribution of any causal responsibility for the problem. Instead, the burdens of moral agents should be distributed according to criteria such as economic capacity, scientific know-how, and the climate management capacity of individuals and social, political, and economic institutions. This leads to various specifications of the principle depending on the context and pillar of climate governance. In climate mitigation, it is relevant that polluters can secure a decent living standard. In climate adaptation, economic capacity and know-how are more key. Indeed, we might even argue that pollution is not relevant at all for assigning responsibilities for climate adaptation (Wallimann-Helmer, 2016; Wallimann-Helmer et al., 2019)

These examples show why specifying and weighting such principles for the various governance levels of climate action and pillars of climate policy allow context-sensitive assessments of the various ethical implications and challenges. A closer look at a specific example illustrates this claim. In the Seti basin in the Himalayas of Nepal, local communities risk heavy flash floods due to landslides that regularly block the river. This risk is increased by changing climatic conditions, which make debris flows more probable. However, although it seems obvious that industrialized countries bear the heaviest responsibilities for these increased risks, the implementation of local adaptation action depends on the capacities of local governments and communities. Consequently, considerations of ability to pay may become more important when analyzing how to differentiate responsibilities than considerations about contributing to the problem or profiting from such contributions either in the past or today. Moreover, in the Seti basin, local communities were relocated but then voluntarily moved back to flood risk zones from the land provided by the local government. Consequently, their responsibility in exposing themselves to flood risks once again also requires assessment by other specifications of core principles. It might be said that these communities now become the ‘polluters’ bearing at least some responsibility for their self-imposed exposure to higher risks.^3^

Together with our discussion of some prominent principles of climate ethics above, this example illustrates how the principlist methodology makes ethical principles specific to the context in two steps. First, specifying principles requires an interpretation of more abstract principles for a given context by identifying which stakeholder should do what for whom and how. However, the principlist methodology assumes that the principles of the core set have no a priori justified ranking. Second, weighting principles is a way of clarifying their ranking in a particular context. This can mean that even if two contexts concern climate adaptation, the same principles cannot necessarily be used to decide the case and that even the same principles might lead to different implications if they are specified differently. We believe that this is a strength of principlism. It relies on the formulation of a theoretical framework which is clear at the normative and prescriptive levels but allowing for specifications and weighting of principles according to context and area of climate governance.

However, principlism has been criticized for several reasons. First, due to its reliance on a set of specific principles, it constrains ethical deliberation and risks ignoring the moral parameters relevant to a given situation. Second, even if it aims to define principles relevant only to a specific context of practice, once the set of principles is defined, they are specified and weighted in a top-down manner similar to conventional approaches in climate ethics; they thus risk remaining insufficiently context specific. Third, even though we can try to define a core set of principles from climate ethics research, it is difficult to know whether the chosen set of principles optimally captures the interests, values, rules, principles, goals, and priorities that emerge from the ethical deliberations of the stakeholders in a given context. In addition to the principles discussed above, other principles may also be relevant. For example, some authors claim that it is more important to consider the equal distribution of emission rights or rights to assistance in climate adaptation and the distribution of risks (Wallimann-Helmer, 2019b, 2021). Furthermore, empirical environmental justice research tends to argue that in addition to claims of distributive justice, recognition and procedural justice are also crucial elements of ethical deliberation (Huggel et al., 2016).

These are reasons why we believe that a principlist approach must be complemented by environmental pragmatism. In the next section, we argue that this is required for three reasons. First, environmental pragmatism provides a methodology for grasping all the relevant facts and empirical details about any context that demands ethical scrutiny. Second, by gathering all the information relevant to a moral context, it also offers an interpretative method for specifying and weighting the principles to be applied. Third, pragmatism also provides resources for institutionalizing and organizing ethical decision-making by involving all stakeholders, including the most vulnerable and disadvantaged as well as those on the margins of power.

### 3.2. Environmental Pragmatist Ethics

Environmental pragmatism constitutes a philosophical movement that emerged around the 1990s in response to the shortcomings on practical policy issues of many academic contributions within environmental philosophy (Light & Katz, 1996). This approach to environmental ethics pursues two complementary aims. First, it offers a practical contribution to the development of environmental policy beyond the ontological debates that characterize environmental ethics.^4^ Second, it proposes a pluralistic moral stance capable of integrating the entire environmental community to engage a diversity of actors and disciplines in discussions of public environmental problems that demand ethical scrutiny. Various interpretations of environmental pragmatism have attempted to address these aims by offering versions of pragmatism that are either more strongly practice-oriented^5^ (Kowarsch & Edenhofer, 2016; Light, 2011, 2012, 2017; Light & Taraska, 2014) or more theory-driven (Weston, 1985; Parker, 1996; Hickman, 1996, and see more recently; Fesmire, 2020). Below, we clarify key elements of our approach to environmental pragmatism to specify areas of compatibility with principlism in climate ethics.

Environmental pragmatism is characterized by its method of inquiry: rather than constituting a philosophical doctrine of its own, it unfolds as an interdisciplinary practice that structures our analysis of problems in the world. Environmental problems are approached according to their own experiential modalities instead of using them as ‘props’ for philosophical debates, as Jamieson (1990) would say. In this situationist perspective, experience precedes any formalized theoretical construction. In other words, pragmatist environmental investigation focuses on the plurality of values and ethical concepts emerging from situations rather than on the top-down application of normative theories and principles.

Pragmatists’ main criticisms of the conventional normative theories of consequentialism, deontology, and virtue ethics concern their conception of normativity, which usually distinguishes the normative from the empirical. Instead, pragmatists argue that the normative and the empirical are strongly interconnected (Hache, 2011). In this approach, a normative evaluation of an ethical challenge constitutes a social theory of its own that cannot be decontextualized and is deployed from a variety of social and moral terrains and from complementary disciplinary resources. From this pluralistic perspective, environmental actions are best decided in participatory and democratic mechanisms involving the state, civil society, and industries.

For instance, an increase in the frequency and intensity of debris flows and rockfalls in recent years has motivated local communities in Swiss mountain regions, such as Guttannen in the Bernese Oberland, to plan climate change adaptation measures.^6^ Here, in a case of climate change adaptation in which specific regions and communities face negative climate impacts, environmental pragmatism offers methods for structuring deliberative decision-making. The pragmatist approach emphasizes the importance of investigating the plurality of value judgments to best justify and prioritize climate action in local governance contexts.

This may mean engaging with various regional stakeholders to develop and institutionalize climate change adaptation in a participatory process using, for example, a ‘world café’ format (Steinemann et al., 2016). Such a format invites the regional stakeholders to identify and assign responsibilities for the development of concrete measures supporting the various views, values, and norms in the case under scrutiny. A pragmatist approach requires that ethical assessment must involve all stakeholders in a deliberative inquiry. In this sense, it complements a principlist methodology that is chiefly a tool for individual reflection. In our view, implementing an ethical approach by involving all concerned resonates better with real-world conditions than an ethical framework that only serves as a tool for either individual reflection or theoretical investigation by armchair philosophers.

In pragmatism, a normative framework for decision-making has to be refined to fit the actual context and how the context develops over time. Which tools and traits are relevant for prescriptive assessments depends on context and cannot be decided entirely beforehand. Consequently, due to its pluralistic and context-sensitive nature, principlism can only be understood as one among other tools or methodologies for ethical decision-making. The principles forming the core set of principlism must be understood more modestly as working hypotheses to be validated, revised, and potentially modified in the course of collective inquiries rather than as pre-defined principles forming common morality in a context of practice.

In consequence, environmental pragmatism is much more flexible than a principlist approach. Its method of inquiry can grasp aspects of a context that extend beyond the framing that a fixed set of principles can provide. To best appreciate moral experience, environmental pragmatism emphasizes the necessity of examining other components of moral life, such as desires, beliefs, attitudes, values, motivations, consequences, objectives, ideals, and methods and structures of deliberation. This broad examination helps to capture the subtleties encountered in the process of reasoning on situated domains of climate justice. In pragmatism, these ethical dimensions require diverse facets of climate change justice to be addressed. For instance, in the case of a specific adaptation, these concern the climate impacts at stake, the level of risk involved, the estimated probability of occurrence of hazards, and their potential consequences.

Furthermore, the critical function of philosophy is crucial to most pragmatist investigations on environmental policy-making to prevent the pitfalls of an ethics driven by economic and political powers or other vested interests. In this respect, key questions must be asked: Who has the power to act in the situation, and what kind of power is implied: domination, persuasion, influence, or collaboration? What are the deliberative and decision-making structures involved? Are all relevant stakeholders included equally in the discussion and, if not, what are the differences, and do they hinder the ability of some to speak out? Depending on the particular situations, the answers to these questions may differ in one way or another, thus indicating the potential conflicts between agents, institutions, and their various powers.

Once critical scrutiny is brought to bear on the diverse deliberative considerations in an ecological community, pragmatism suggests that it may then be possible to implement a credible moral pluralism that goes beyond the dominant conception of unlimited economic growth and the whims of those in power. This requires that all stakeholders can add their voices to public debates and participate meaningfully in shaping fair climate policies.

By relying on inclusive deliberative processes, environmental pragmatism can further support the specification and weighting of norms and values because they provide empirical details to a case and its ethical challenges through the perspectives of stakeholders that might easily be overlooked (Milot et al., 2015; Minteer, 2012; Minteer & Collins, 2005a, 2005b, 2010; Norton, 2015). To support this deliberative process, environmental pragmatism draws on methodologies from the social sciences and from a variety of complementary disciplines to better understand a specific context under ethical scrutiny. In a concrete case, pragmatism marshals methodological and theoretical knowledge to fit the particular contexts of climate action and governance (Voisard, 2020; Voisard et al., 2020). This empirical sensitivity of pragmatist methods is an important complement to a principlist methodology in specifying and weighting principles relative to contextual specificities and local priorities, such as safety, accessibility of resources and infrastructure, and quality of life.

The strength of a pragmatist approach clearly lies in its methodological flexibility and its adaptive capacity. This strength is scarcely negligible for a discipline such as climate ethics that seeks rigorous theoretical anchors that are yet sufficiently flexible to be engaged with environmental practices and real-world conditions. However, from a classical normative ethics perspective, the flexibility of this approach becomes its weakness, because it implies a lack of clarity at the prescriptive level. What is lacking is a clear and simple normative approach to guiding decision-making. This is what principlism can provide. Principlism helps to clarify the normative implications of climate governance in particular contexts. Hence, environmental pragmatism provides a structure for ethical deliberation and for gathering all relevant context-sensitive information, and principlism provides a tool for deciding the ethical conflicts emerging from such an inquiry.

## 4. Common Ground in Principlist and Pragmatist Methodologies

In the previous sections, we criticized the methodologies used by three common normative approaches to climate ethics. We then presented two approaches to climate ethics that, when properly combined, can address the shortcomings of traditional normative approaches. Here, we clarify how our combined approach to climate ethics is characterized on methodological grounds. We argue that despite differences, principlism and pragmatism share common ground in their methods of ethical reasoning. This is why their potential to complement each other is much greater than possible conflicts between them.

We have argued that top-down approaches that use deductive-only reasoning are problematic in local climate governance. This approach of deductive reasoning has two alternatives. The first approach relies on inductive reasoning for ethical justification. By comparing cases, it seeks to develop rules for solving ethical challenges of similar structure. Certain forms of virtue ethics might base ethical justification on bottom-up reasoning of this kind. But it is arguably one of the oldest ethical methodologies, casuistry, that best embodies this type of ethical reasoning. Casuistry typically starts from paradigmatic cases to formulate general moral rules. By analogical reasoning, it then tries to infer what would be morally correct behavior in less paradigmatic cases (Paulo, 2016).

However, although casuistry is not yet developed in climate change ethics literature, we can expect it to fail to convincingly meet the criticisms we have formulated of the conventional approaches to climate ethics. This is because it tends to generalize from paradigmatic cases to analyze the new challenges of various contexts with their own specificities without being sensitive to these. For example, when applied to contemporary emitters as a paradigmatic case, the polluter-pays principle does a very good job in identifying those who need to reduce their emissions the most. Although such an approach may apply to emitting contexts, it is far from clear that applying this principle by analogy will suffice to inform and decide proper measures for each pillar of climate action and governance in all cases.

For instance it is of no help to local communities in need of adaptation to inform them that high emitters should provide the most support when the challenge they face is weak governance structures and lack of resources. What is crucial is that they are helped efficiently and effectively by those who can best assist (Huggel et al., 2016; Wallimann-Helmer, 2016). Hence, even though for climate governance it makes sense for climate ethics to rely on the polluter-pays principle in the most paradigmatic cases, such an inference by analogy cannot always be generalized. This favors principlism over casuistry as a methodology that would weight the decisive importance of the polluter-pays principle differently depending on context.

We believe that the method of ethical reasoning underlying principlism and pragmatism can supplement the shortcomings of the methodological models discussed thus far. Principlism typically relies on the method of reflective equilibrium as formulated by Rawls (1999). This model rests on ethical reasoning that does not rely exclusively on deductive top-down approaches nor exclusively on inductive bottom-up approaches. Instead, the method of reflective equilibrium relies on a sequence of inductions and deductions as we work back and forth, adjusting our moral beliefs to achieve balance in ethical reasoning. This sort of reasoning bears a striking resemblance to the pragmatist logic of inquiry (Dewey, 1938), which also seeks coherence and revision among specific judgments and general statements whilst relying on empirical descriptions to support ethical deliberation processes. Unless we adopt an overly narrow conception of pragmatism and principlism, this method of ethical reasoning offers a key resemblance between the two (Schmidt-Felzmann, 2003). This makes both approaches perfect complements united in a more context-sensitive and practice-relevant method for climate ethics.

Certainly, principlism and pragmatism’s methodological models present several distinctions and potential conflicts. However, these are arguably less fundamental than subtle when guiding the concrete policy decisions of local climate governance. For brevity’s sake, we can only hint at two potential tensions between the two models. First, principlists might be reluctant to simply follow coherentism as a methodology of ethical justification. Coherence theory suggests that no core set of ethical principles guides ethical reasoning in a given context of practice and governance, which is the core assumption of principlism (Beauchamp & Childress, 2013). Pragmatists, by contrast, do not necessarily endorse coherentism but certainly reject all claims that may appear to be associated in some way with epistemological foundationalism, a philosophical conception in which certain beliefs or sets of principles, as in principlism, are claimed to be fundamental to a given quest for knowledge. Hence, whilst pragmatism is rather critical of this type of quest for philosophical systematization, principlism does not intend to dissociate itself from such a quest. However, what pragmatism and principlism do share is the embedding of ethical reasoning within contexts of diverse value judgments and making it coherent with these.

A second distinction between pragmatist and principlist methodologies lies in the starting points of their ethical reasoning. For pragmatism, experience comes first: reasoning begins by induction. In contrast, principlism is more inclined to begin ethical reasoning by specifying principles: reasoning begins by deduction. But in neither approach does ethical reasoning stop there. It continues through a series of adjustments and verifications that draw on the empirical and theoretical elements encountered in open-ended ethical deliberation and political decision-making. Hence, even though their starting points may differ, both approaches share the idea that ethical reasoning must not only be coherent with other moral values but also achieve an equilibrium between normative principles and the empirical specificities of a given case. Such a balanced method of ethical reasoning is required to engage appropriately with the complexities, concrete subtleties, and the messy nitty-gritty of local climate governance. In practice, it seems irrelevant to emphasize the extent to which this deliberation starts from principles to be specified or from empirical facts.

## 5. Conclusion

The objective of this paper was to begin articulating a methodological framework of climate ethics for practical reflection and decision-making in climate change governance. We have criticized the methodologies commonly used in climate ethics. We have argued that deontology and consequentialism use classic top-down methodologies to deduce prescriptive conclusions mechanically without being sufficiently context sensitive and flexible to guide ethical decision-making appropriately in concrete climate contexts. In turn, virtue ethics lacks normative guidance and resources for collective deliberation but offers a contextual model for ethical reflection.

We have argued that a more practice-oriented climate ethics can be obtained by articulating key features of principlism as the methodology of mid-level principles and environmental pragmatist ethics. Principlism provides a contextualizable normative framework for environmental practitioners, whereas pragmatism offers an ethical deliberative framework that is faithful to the richness and the complexity of collective moral experience. Both methodologies can be interpreted as midway between bottom-up and top-down reasoning, in which pragmatism falls closer to bottom-up approaches and principlism closer to top-down ones. We have argued that, when properly combined, they can help address the methodological shortcomings of classical climate ethics approaches. The purpose of this article was not to develop ethical decision-making instruments but to investigate a novel methodological framework. In our view, joining pragmatism and principlism appropriately can reach the right balance in ethical reasoning to guide ethical decision-making in concrete local climate governance contexts. This form of reasoning is sufficiently flexible to resonate with real-world conditions and support normative environmental decision-making by providing a contextual and pluralistic ethical framework for guiding climate action and governance.

## Notes

1. Deontological ethics is an approach in moral philosophy that focuses on rules, norms, and principles. However, the principles of climate justice can be articulated from a variety of ethical perspectives. It is possible to ground general principles of climate ethics on the autonomy of moral agents, just as it is possible to ground these principles on a utilitarian conception of individual well-being (Broome, 2012).

2. For an example of such an initiative, see https://adaptationmemphre.ca.

3. For more details on this case study in Nepal, see https://knowledgeforclimate.net.

4. According to environmental pragmatists, the theoretical commitment to value monism of many environmental philosophers tends to exclude them from practical and environmental policy-making circles, whose views are often pluralistic.

5. It is worth noting that one reading of environmental pragmatism has been articulated in arguably the most important philosophical contribution to climate diplomacy to date (see Light, 2017).

6. For more details on this case study in Guttannen, Switzerland, see https://knowledgeforclimate.net/.
